# A novel SpaSA based hyper-parameter optimized FCEDN with adaptive CNN classification for skin cancer detection

**DOI:** 10.1038/s41598-024-57393-4

**Published:** 2024-04-23

**Authors:** Rizwan Ali, A. Manikandan, Rui Lei, Jinghong Xu

**Affiliations:** 1 Department of Plastic Surgery, The First Affiliated Hospital, School of Medicine, Zhejiang University, No. 79 Qingchun Road, Hangzhou, 310003 China; 2 Department of ECE, SRM Institute of Science and Technology, Kattankulathur, Chennai, 603 203 India

**Keywords:** Skin cancer, Malignant melanoma, Sparrow search algorithm, Adaptive CNN, Dermoscopic images, Fully convolutional encoder–decoder network, Medical research, Biotechnology

## Abstract

Skin cancer is the most prevalent kind of cancer in people. It is estimated that more than 1 million people get skin cancer every year in the world. The effectiveness of the disease’s therapy is significantly impacted by early identification of this illness. Preprocessing is the initial detecting stage in enhancing the quality of skin images by removing undesired background noise and objects. This study aims is to compile preprocessing techniques for skin cancer imaging that are currently accessible. Researchers looking into automated skin cancer diagnosis might use this article as an excellent place to start. The fully convolutional encoder–decoder network and Sparrow search algorithm (FCEDN-SpaSA) are proposed in this study for the segmentation of dermoscopic images. The individual wolf method and the ensemble ghosting technique are integrated to generate a neighbour-based search strategy in SpaSA for stressing the correct balance between navigation and exploitation. The classification procedure is accomplished by using an adaptive CNN technique to discriminate between normal skin and malignant skin lesions suggestive of disease. Our method provides classification accuracies comparable to commonly used incremental learning techniques while using less energy, storage space, memory access, and training time (only network updates with new training samples, no network sharing). In a simulation, the segmentation performance of the proposed technique on the ISBI 2017, ISIC 2018, and PH2 datasets reached accuracies of 95.28%, 95.89%, 92.70%, and 98.78%, respectively, on the same dataset and assessed the classification performance. It is accurate 91.67% of the time. The efficiency of the suggested strategy is demonstrated through comparisons with cutting-edge methodologies.

## Introduction

**Figure 1:**
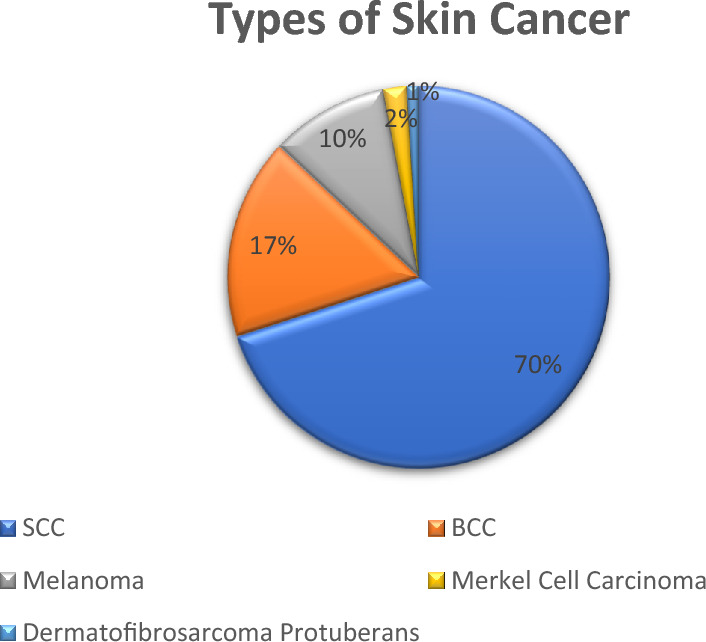
Showing the ratio of different varieties of skin cancer increasing. SSC (Squamous Cell Carcinoma), BCC (Basal Cell Carcinoma), Melanoma, Merkel Cell Carcinoma, and Dermatofibrosarcoma Protuberans.

In certain nations, skin cancer is a disease that is on the rise. If detected early, this kind of cancer is treatable^1^. Therefore, reducing skin cancer mortality by early identification is a potential technique^2^. Figure 1 shows an increase in all types of skin cancer^3^. To attempt to predict skin cancer diagnoses with a margin of error that is lower than that possible in humans^4^ since there are several potential dangers associated with them, in addition to cost and morbidity. Minor mistakes happen in such systems, and diagnostic accuracy is not always adequate. Consequently, a powerful computer-aided diagnostic system can benefit physicians in avoiding misdiagnosis. Preprocessing, segmentation, feature extraction, and classification are a few processes in a typical method for early skin lesion identification^5^.

Because the result of each phase acts as the input for the following step, each step is crucial to avoiding misdiagnosis. Preprocessing, a preliminary step in computerised cancer detection, significantly affects the reliability of the findings^6^. Preprocessing is essential for the success of such systems^7^, but thus far, only a few studies have discussed preprocessing methods.

The preprocessing processes for detecting skin cancer may be broken down into image enhancement, image restoration, and artefact removal. A distinct approach is used in each phase, as explained in this white paper. Furthermore, the follow-up strategy selected for the automated system affects the selection of the preprocessing method. The most popular preprocessing methods are Gaussian, mean and median, and speckle noise filters^8^.

Tumors and abnormalities in the lymphatic system or blood can result from aberrant skin cell division. Both benign and malignant swellings can develop through the body’s lymphatic system, whereas benign swelling is localized and does not spread far^9^. Since skin cancer can be seen with the unaided eye, it is easier to detect than other malignancies.

Exposure to ultraviolet (UV) radiation from direct sunshine or chemicals released by specific types of light bulbs are the two leading causes of skin cancer. The DNA of the aforementioned cells is altered by these two substances, which changes how the cells grow and evolve and causes them to become malignant tumours^10^.

Deep learning techniques have become important as an image processing tool in recent years. Due to its automated feature recognition, enhanced prediction methods, and classification skills, deep convolutional neural networks (CNNs) are becoming more and more prominent in computer vision applications. However, using CNN architectures for image classification does not solve segmentation issues. Standard CNN’s fully linked hierarchies entirely disregard structural information and generate class probability values, which may result in subpar segmentation outcomes since semantic segmentation emphasizes pixel-level categorization. In a subsequent research phase, fully convolutional networks (FCNs) were developed to swap out CNNs’ completely connected layers with convolutional and deconvolutional layers in order to enhance pixel segmentation performance^11^. Reduced network parameters and accelerated training are achieved by avoiding dense hierarchies. The FCN design typically consists of convolutional layers, pooling layers, rell layers, and one pooling layer. While a single pooling layer is used to up-sample the output of the final down-sampling layer to the input size, convolutional and pooling layers are helpful for down-sampling image features. The model’s performance may be determined by contrasting the image ground truss with the unspooling layer’s output. Performance is constrained when only one up-sampling layer is used (this FCN variation is promising but cannot be taught).

This work suggests another FCN variation that includes encoder and decoder components to enhance the performance of pixel-level segmentation (FCEDN). Convolutional, max pooling, and dropout layers make up FCEDN’s encoder for the crucial down-sampling step required to extract feature maps. To recover the functional map resolution, the network also incorporates a learnable decoder into the up-sampling process. The decoder samples the pre-convolutional, pooling, and dropout layers of the encoded output one at a time. The decoded result is an output layer with measurements that perfectly match those of the input image. Due to its trainable encoder and decoder, FCEDN performs better than FCN with a single up-sampling layer that is not trainable.

One of the most crucial imaging techniques for identifying and categorising skin tumours is dermoscopy. These produced images are amenable to automated analysis that aids dermatologists in making wiser choices. The latter is a database that allows the patient’s best course of action to be chosen. This study can be facilitated by new techniques like complex product neural networks (CNN)^12^. The classification of different forms of skin cancer and other skin illnesses using CNN technology has achieved a “professional level”^13^.

Complete medical systems are being constructed in practice using contemporary methods like deep neural networks and machine learning techniques^14^. This new method’s significance stems from its capacity to identify patterns, which is crucial in the medical industry. For instance, when paired with World Cup optimization techniques, neural networks like Multilayer Perceptron (MLP) and Artificial Neural Networks (ANN) are particularly good at detecting melanoma in images^15^.

Additionally, several researchers have used MLP-ANN with grey wolf optimization to detect melanoma efficiently and accurately^16^. Similar results were obtained when a CNN was optimised using the Satenbauerbard optimization technique^17^. As a result, it is evident that computer-based techniques and machine learning algorithms significantly positively influence on data mining techniques, logistic analysis, and accessibility choices^18^. Additionally, with straightforward data management, robots and computers may do the same work in less time^19^.

The key contributions of this challenge are:

- The primary objective of this research project is to create an adaptive CNN model that can accurately and automatically categorize different types of skin cancer into melanoma and non-melanoma categories. The classifier is trained with initial weights that are fixed, and the misclassification error is computed using a weighting system.
- Preprocessing the image data, which includes things like image resizing, color space conversion, contrast improvement, noise reduction, and hair removal, is essentially what the first phase entails by using Adaptive Histogram Equalization and Adaptive Median Filter.
- In this work, a threshold-based automatic approach for skin cancer detection, classification, and segmentation utilizing a meta-heuristic optimizer named sparrow search algorithm (SpaSA) is proposed with fully-convolutional residual encoder-decoder-based neural network architecture. The major contribution of this work is in the unique design of the encoder–decoder network that makes it especially suitable for skin cancer segmentation.
- In this work, a threshold-based modified approach for skin cancer disclosure, classification, and division utilizing a meta-heuristic optimizer named sparrow see calculation (SpaSA) is proposed with fully-convolutional remaining encoder–decoder-based neural organize plan. The major commitment of this work is inside the one of a kind arrange of the encoder–decoder organize that makes it especially fitting for skin cancer division.
- The datasets used in this work are from the common PH-2, ISIC-2017 and ISIC-2019 challenges, which have various image resolutions and problems with class imbalance. To employ the transition learning approaches, which automatically adapt the depth, breadth, and resolution of the network and learn more intricate and fine-grained patterns from lesion images, to address these two problems and attain great performance in classification.
- To precisely define melanoma lesions, use segmented skin lesions. In order to categorize each skin lesion into a benign or malignant category, the features that were collected from the skin lesions are then entered into the feature classification module.

The structure of the paper is as follows. Skin cancer is covered in “Introduction” section. The summary of the study is given in “Literature survey” section. The dataset for skin cancer is shown in “Dataset” section. After that, in Chapters 3, 4, and 5, several techniques are proposed for preprocessing, segmentation, and classification, and the most successful methods are described based on the literature. The paper’s conclusion is found in the final part.

### Motivation

Experience and exact skills may be necessary for an accurate diagnosis of various illnesses in order to increase diagnostic accuracy. As a result, having advanced equipment that can deliver precise diagnostic results, superior therapies, and fewer patient biopsies is crucial for dermatologists. Both medical professionals and patients may find it simpler to diagnose certain ailments with the aid of deep learning. As was already noted, using these technologies can save doctors much time and work, but the primary objective is undoubtedly correct diagnosis.

## Literature survey

The area of image processing known as semantic image segmentation aids in determining the size and form of items in an image. The field of image segmentation using FCNs is currently advancing quickly. Several FCN-based image segmentation networks have been published in the literature. Using Skip Architecture, Long et al. merge semantic and shape information in deep and coarse layers. Semantic segmentation is proposed using an FCN model. Another network, known as the U-Net design, employs symmetrically expanding and contracting routes, giving it the appearance of a U-shaped architecture.

The contract route involves repeated convolutional and pooling layers with doubled feature channels at each down-sampling level to pass contextual information to high-resolution layers. On the other hand, the augmentation procedure uses up-convolutional layers that account half of each layer’s feature maps to locate the precise position. It produces good splits by eliminating linked layers and simply using the critical portions of each convolution. The FCN model suggests dense pixel-level categorization of images.

The suggested VGG-16 network generates the network; however, the final layer’s initialization is random. From remote sensing data, the model is used to extract footprints automatically. To discover that, in terms of pixel-wise segmentation performance, our elaborate FCN architecture, SegNet, outperforms FCN, DeepLab-LargeFOV, and DeconvNet. Encoders and decoders are both used in the SegNet architecture. Although the encoder architecture comprises 16 convolutional layers, the decoder design inverts the low-resolution encoder function to the entire input resolution function for pixel-level classification. These three ensembles are suggested to handle the segmentation problem of pleural nuclei after optimising FCN, U-Net, and SegNet, and it has been demonstrated that they are more efficient than each individual and majority voting approach^20^. The new F2FCN introduces functional adaption modules in conjunction with applicable reuse. The feature fitting module eliminates potential noise and ensures that several feature levels are correctly fused, while the feature reuse module retrieves features from numerous levels. The network includes symmetric increasing links like U-Net and decreasing paths for effective hepatocellular carcinoma diagnosis. Using FCN and CNN, created a completely automated computer-aided diagnostic (CAD) system. The VGG-16 model is the foundation for this CAD system’s FCN architecture, which includes two extra “jumping structures” for segmenting the liver and tumour. A 9-layer CNN is given the raw and segmented images for HCC classification. They suggested an additional model that combines SegNet and FCN-8 for accurate segmentation of images of plantar pressure^21^.

For the localisation and categorization of skin cancer, Ref.^22^ presented an ensemble architecture. The image is first preprocessed, resized, and segmented using Otsu’s approach utilising a bioorthogonal 2D wavelet transform. Then, to extract features, utilise VGG-16 and pretrained AlexNet. Studies have demonstrated that it performs better than other approaches in terms of accuracy.

To correctly discriminate between benign and malignant skin lesions, Ref.^23^ created a deep CNN model. They used transfer learning to fit this model into another model. The suggested approach has a training accuracy of 93.16% and a testing accuracy of 91.93%, making it quicker, more dependable, and more robust.

For the first time, non-melanoma skin cancers were segmented and classified by an interpretable deep-learning system that used many classes^24^. They divided the tissue into 12 dermatological classes to characterize it. These classifications include skin-stratified layers, sweat glands, and hair follicles. High accuracy (> 93%) was attained when categorizing the whole tissue. This technique may be utilised to carry out everyday pathologist activities like assessing the surgical margin gap.

New data classified privately using mobile device technology is reported in Ref.^25^. An on-device inference program specially trained for classification is used to carry out the classification procedure. This work mainly addresses skin cancer, one of the most prevalent human malignancies, does a case study to assess the system’s effectiveness, and outlines the project’s main idea.

Reference^26^ suggested a method to determine whether test samples contain melanoma. The following stages are discussed in this paper. The preprocessed images are merged and utilised to gather labelled data before pixel extraction. The extracted pixel intensities are gathered in a database-compatible array that may be used to store them. Using a handy kernel, SVM with labelled data correctly categorises samples using previously learned data. 90% categorization accuracy is demonstrated using the suggested system.

Reference^27^ proposed a technique for automatically identifying face skin diseases using a pre-trained deep CNN. Their algorithm identified eight distinct face skin disorders with an accuracy of 88 percent. Utilizing specific image preparation methods, they redesign the images. The database size is then increased by gathering and resizing images from various sources. Images that have undergone considerable processing are utilised as training and additional validation sets.

Reference^28^ developed a novel method employing deep learning methods, such as computer vision systems, to detect many skin problems automatically. The system architecture uses the vote results of three publicly available image recognition designs to categorise different forms of skin diseases (InceptionV3, Inception ResnetV2, and MobileNet). As a result of the implemented model’s rigorous training to recognize up to 1000 different groups, the system’s accuracy is high. This approach uses feature extraction, training, and testing procedures like most other methods.

Reference^29^ employed a two-step method to identify clinical histology instances, including computer vision and machine learning. The dermatological images were initially preprocessed and feature extracted using several methods. They then used machine learning algorithms to categorise illnesses using the data.

### Problem statement

To prevent these debilitating treatments and obtain effective treatment, melanoma treatment includes chemotherapy and radiotherapy. Early diagnosis is among the most efficient solutions. Several CAD systems are currently available for the identification of pigment skin lesions, such as Dell’Eva-Burroni Melanoma Image Processing Software, which gives a low performance in actual implementations. However, general conclusions about the performance of these systems are hard to define. In organised research, the multiple image acquisition techniques such as dermoscopic, clinical, and normal camera images further complicate the classification task in one global methodology. Therefore, the new CAD programmes are also far from ideal and require further advances to enhance melanoma detection and diagnosis. In addition, two significant problems are posed in the classification of skin pigment lesions into malignant and benign cancer.

Chemotherapy and radiation therapy are part of the melanoma treatment process to avoid these ineffective therapies and receive appropriate care. The most successful treatment is early diagnosis. Several CAD systems, such the Dell’Eva-Burroni Melanoma Imaging Software, are already available for detecting pigmented skin lesions; however, their performance could have been in better actual applications. Drawing broad judgments about how well these systems function is challenging. Multiple image acquisition methods, such as dermoendoscopy, clinical imaging, and general camera imaging, complicate the categorization process in planned investigations from a global perspective. Therefore, more improvements are required to enhance melanoma detection and diagnosis, as current CAD algorithms are unsuitable. Classifying pigmented skin lesions into dangerous and benign malignancies raises two significant problems.

Precision segmentation is a complex process in enhancing the detection of aberrant lesions and the differentiation between benign and malignant lesions.

In order to categorise pigmented lesions into benign and malignant skin malignancies, the second step is to collectively extract the most discriminative elements that define the relevant categorization.

## Dataset

The efficiency of the proposed approach is assessed using three simulated datasets of dermoscopic skin lesions.

**Figure 2:**
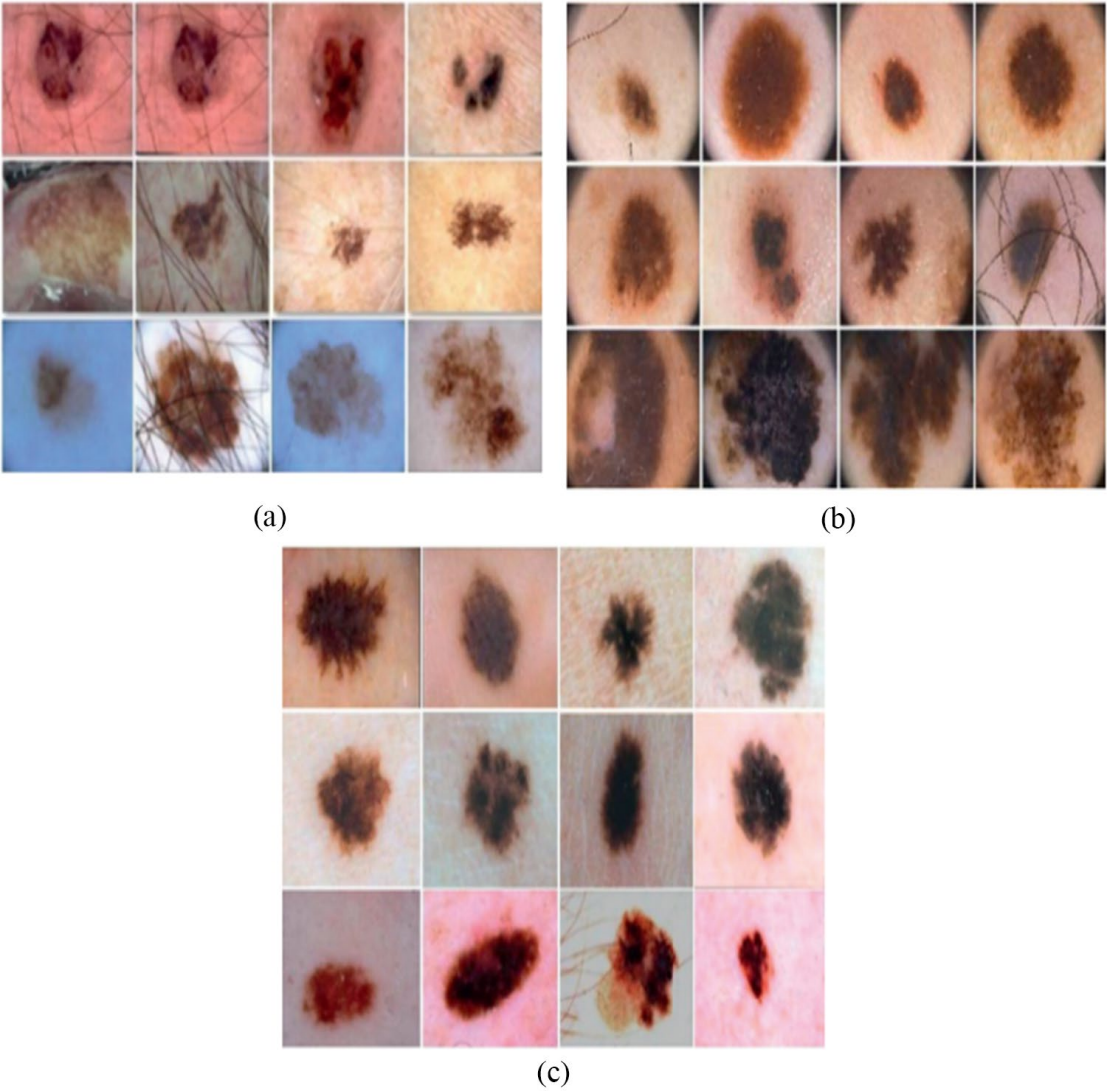
Three examples of dermoscopic images are from the ISIC-2019 data set, PH-2 data set, and ISIC-2017 data set.

To detect melanoma, the ISIC-2019 dataset uses a dermoscopic image format in conjunction with skin lesion analysis. Dermoscopy is an imaging technique that eliminates surface reflections from the skin. It contains samples from the ISIC-2019 dataset and enhances the accuracy of the diagnosis (see Fig. 2a).

### PH-2

It is a dermoscopy skin lesion information database. For clinical diagnosis and research, the PH-2 collection includes a sizable number of passive skin lesion segmentation images. Dermatologists or skin experts recognize different dermoscopic structures of dermatological lesions. Scientific studies will frequently use the PH-2 Dermoscopy Image Dataset and the PH-2 Dataset Dermoscopy Image Samples (see Fig. 2b).

### ISBI-2017

A collection of more than 10,000 images of dermoscopic skin lesions for use in scientific study and medical diagnostics. Dermoscopic images of a few skin lesions with annotations and labels from a recognised skin cancer specialist. Figure 1 displays dermoscopy images of representative skin lesions from the ISIC-2017 dataset (see Fig. 2c).

### Proposed method

There are several resolutions available for skin cancer images, including 1504 $\times$ 1129, 1022 $\times$ 767, 9441 $\times$ 127, and 767 $\times$ 576. The segmentation performance might not live up to expectations due to the enormous volume of image input without video preprocessing and the size of each images. It might take a while to diagnose skin cancer via education. Bilinear interpolation is used to make training and test images more manageable in size while keeping the aspect ratio before being fed into the model to address these problems.

### Skin cancer detection systems pre-processing

**Figure 3:**
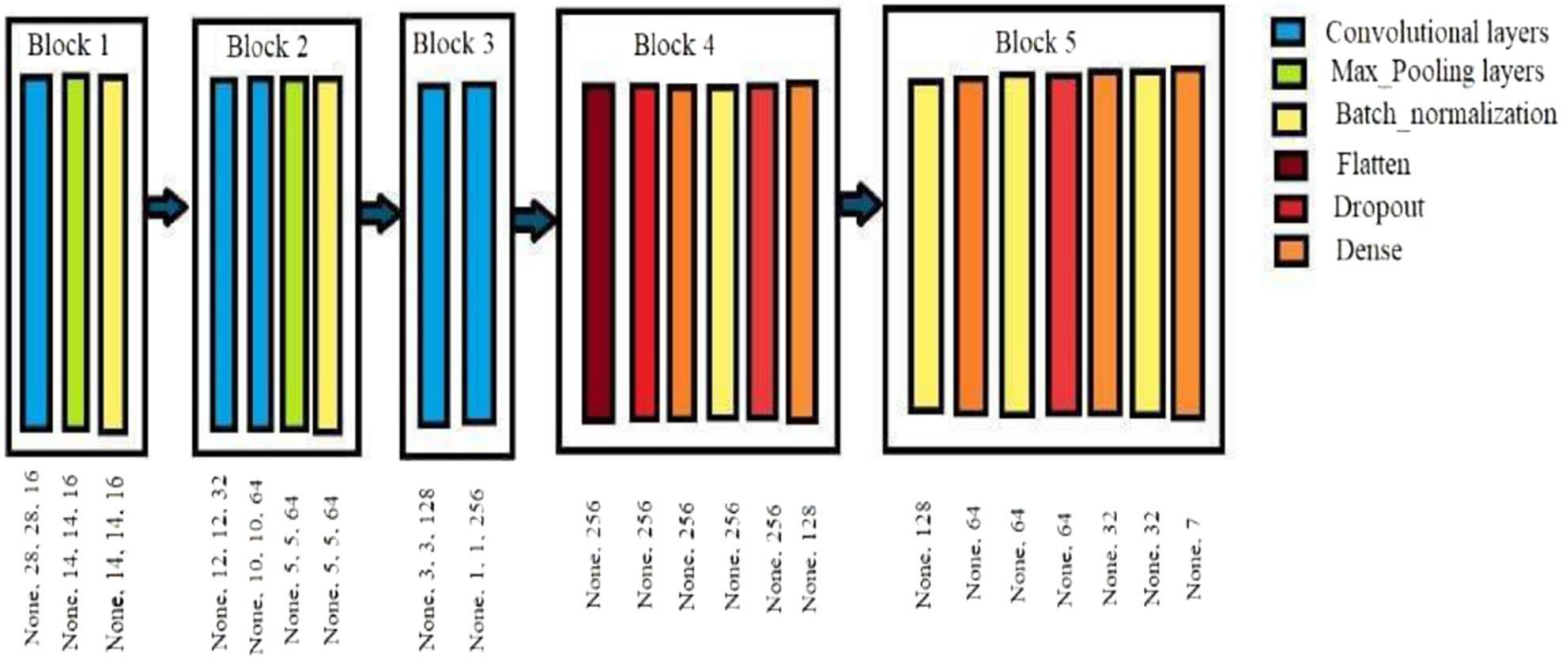
Adaptive convolutional neural network architecture.

A crucial detection component is image preprocessing, which can improve the original image’s quality by removing noise. It must be used to look for abnormalities with minimal influence on the outcomes^8^. The primary goal of this stage is to enhance the quality of melanoma images by eliminating extraneous and superfluous background elements that will not be used in subsequent processing. Selecting the proper preprocessing method can significantly increase system accuracy^9^. Figure 3 depicts the general technological architecture used during the preprocessing step for medical images.

The preprocessing step’s objective may be achieved by three processing phases: image enhancement, image restoration, and hair removal. This article thoroughly explains the technique above for researchers working in the preprocessing stage of automated detection.

#### Image optimisation

Image enhancement is a crucial stage in enhancing an image’s aesthetic appeal. An extra auto-detection phase is a source of “better” modified representations^10^. Thus, there are three different types of image augmentation.

#### Image scaling

Image scaling techniques are used since there is no uniform and standardised image size. The first stage in resizing skin cancer images so they have a constant pixel width but a variable height resizable^11^ is to account for the fact that they can be gathered from various sources and sizes.

#### Color space transformation

Researchers tried to extract additional complementary colours from images for further processing since colour information is inextricably linked to skin cancer detection systems. Typical colour spaces include RGB, HSV, HSI, CIE-LAB, CIE-XYZ, etc. Red, green, and blue spectral wavelengths make up the colour space known as RGB. RGB is the colour system that is most frequently used in image processing. Different colour space representations were created since the RGB colour space has some modest limits in sophisticated processing.

#### Noise removal

Image denoising is a crucial step in the preprocessing of images. It is incredibly challenging to apply efficient rejection algorithms to otherwise noisy images.

##### Adaptive filter

This filter performs well when noise is constant power additive (“white”) noise, such as speckle noise.

Random noise can be reduced with an adaptive local noise reduction filter.

##### Adaptive median filter

Smooths non-impulsive noise while preserving detail, which is impossible with conventional median filters.

#### Removing thick hairs

Most repair filters can smooth out skin wrinkles and small blood vessels, but the image may still show dense hair. Dense hair has been noted as a frequent barrier that may cause the division process to be misled in automated examination of tiny skin lesions. Researchers employed mathematical morphology approaches to remove thick hair from skin cancer images. The challenge was used to obtain images sans hair. The skin cancer detection system’s preprocessing step results in an image that can be separated from the original image and is almost ready for segmentation.

## Segmentation

### A SpaSA-based hyper-parameter optimized FCEDN model for segmenting skin cancer images

The four fundamental processes of the model are image preprocessing, hyperparameter selection optimization with SpaSA, building and training FCEDN utilising these hyperparameters, and model assessment.

### Creating the FCEDN

The trainable encoder and decoder components are arranged in numerous layers of the deep neural network known as FCEDN. While the transposed convolution, pool, relu, and dropout layers make up the up-sampling portion of the decoder or network, the convolution, relu, dropout, and max pooling layers make up the down-sampling portion of the encoder or FCEDN. Every layer has a unique value. While some decoupling attempts using FCNs have yielded encouraging results, creating the proper foundation for an application-specific FCN is far from simple. Standard layouts suggested in the literature were frequently influenced by earlier work or the results of trial and error. The original FCEDN structure has 4 convolutional layers, 4 relu layers, 1 dropout layer, 2 pooling layers, an encoder, and 4 transposed convolutional layers, which consist of 4 relu and 2 non-pooling layers when considering the associated tasks completed in. a dropout layer for the decoder construction. Convolution, transposed convolution, pooling, and un-pooling layers use kernel sizes ranging from 3 × 3 to 5 × 5.

Additionally, the first layer contains fewer kernels than the following layers, which have between 20 and 200 kernels. To normalise the model, the dropout layer uses a dropout ratio that is thought to be between 0.2 and 0.4. The total number of convolutional, pre-convolutional, pooling, and deconvolutional layers determines FCEDN’s overall architecture. Convolutional over- and under-modelling are caused by changing the number of convolutions, respectively. Except for some functions that require more pools, the same functions can be repeated with fewer pools. Therefore, the number of convolutional layers, transposed convolutional layers, pooling layers, and non-pooling layers in this study is kept between 2 and 10.

### Optimizing SpaSA for FCEDN hyper-parameters

The wolf population update, population initialization, suitability analysis, and FCEDN hyperparameter optimization process are the four processes that makeup EN-FCEDN GWO’s procedure. The convolution kernel size (CV Ks), pre-convolution layer (TCV Ks), convolution kernel size (CV NK), pre-convolution layer (TCV NK), maximum pooling layer (MP Ps), pooling layer kernel size (Un Ps), and DL output layer drop rate (DL) are FCEDN hyperparameters. These parameters are both encoded into a k-dimensional vector during the encoding stage. Within a specific range, the values of the encoded vectors are assumed to be random. Equation (1) gives the ith parameter vector as

1 $${X}_{i}=\left\{{P}_{i1},{P}_{i2},{P}_{i3},\dots {P}_{ik}\right\}.$$

The vector size (k) is 22 if there are 4 convolutions, 2 dropouts, 2 max pooling, 4 pre-convolutions, 2 unrolling layers, and hyperparameters corresponding to various layers. Tcv1 Nk, Tcv1 Ks, Tcv2 Nk, Tc, Tcv Xn, Mp1 Ps, DL1 Dr, Cv2 Ks, Cv3 Nk, Cv3 Ks, Cv4 Nk, Cv4 Ks, Mp2 Ps, DL2 Dr, and Un1 PS. Xi is the I k-dimensional vector of FCEDN hyperparameters, signifying the position. Since FCEDN training is considered while calculating fitness for EN-GWO, investigate if this study has a modest variance in fitness value. Following the creation of the coefficient vector a. Equations (2), (4), and (6) are used to evaluate each agent’s fitness by SpaSA’s A and C^3^. The population update method and the top 3 agents, Xa, Xb, and Xd, are then: Continue as shown in the pseudocode for each iteration that has been specified. The agent with the best FCEDN hyperparameters for building the image segmentation network has the highest fitness value. As a result, the SpaSA goal function’s formula is to maximise the Jaccard coefficient.

2 $$f\left({x}_{i}\right)=\left(\sum_{m=1}^{tim}\left(\frac{\varepsilon +{\sum }_{j=1,l=1}^{j=r,l=c}{y}_{m}(j,l)\widehat{{y}_{m}}(j,l)}{\varepsilon +{\sum }_{j=1,l=1}^{j=r,l=c}{y}_{m}\left(j,l\right)+{\sum }_{j=1,l=1}^{j=r,l=c}\widehat{{y}_{m}}(j,l)-{\sum }_{j=1,l=1}^{j=r,l=c}{y}_{m}(j,l)\widehat{{y}_{m}}(j,l)}\right)\right),$$

where ${y}_{m}\left(j,l\right)$ is the anticipated label represented by pixel $\left(j,l\right)$ in the m-th image of size (rXc) and $\widehat{{y}_{m}}(j,l)$ is the actual value of pixel $\left(j,l\right)$ in the image. The number of images in the training data set is represented by $tim$, and the smoothing parameter e of the position vector ${x}_{i}$ produced by FCEDN is a random value between 0 and 1. Operations that break classes into smaller groups might be more prone to class imbalance. Deep neural networks may reach a segmentation accuracy of 80% by correctly categorising just background pixels. About 20% of the total number of pixels in the image corresponds to segmented sections. Therefore, accuracy may not be the best criterion to use when evaluating the effectiveness of automated segmentation (Kaymak et al.^30^). Therefore, a helpful indicator of segmentation success might be the percentage of redundancy between the observed and predicted masks of the model. It calculates the ratio of the total number of pixels in the expected and measured masks to the shared pixels between the two.

## Classification

### CNN adaptive

The proposed algorithm for the skin lesion classification is the Adaptive CNN architecture. 512 lesion images were utilized as the input size for the investigation. CNNs can handle large datasets because of their architecture. Data augmentation techniques that automatically enlarge lesion images have been deployed to solve this problem. Before the processing stage, the teacher suggests using a CNN with an input of a full-size image of 124 × 124 pixels. The CNN design, which has a total of nine layers, can obtain more training data, which is the most excellent technique to lessen overthinking. Still, the medical imaging lacks labelled data, making it impractical. One of the options is regularization with dropout, which adds two extra product layers to CNN models. Features are extracted using convolutional layers, and the data is down-sampled using pooling layers if a lesion is cancerous or benign.

My network’s brain, efficient, a pre-trained convolutional neural network, extracts characteristics from my melanoma dataset. This network is motivated by Efficient Net’s improved feature extraction efficiency and capabilities, among other factors. Image networks have provided Efficient Net with knowledge in image categorization. Efficient Net was created with Mnas Net as its primary inspiration rather than concentrating primarily on developing the optimum convolutional CNN design. The EfficinetNet-B6 network is made from the baseline network by applying the composite scale g technique, which scales the networks d, w, and r equally using a composite coefficient 0. There are three reasons why EfficientNet-B6 outperforms its rivals. The first network is deeper, captures richer, more complicated characteristics, and generalizes well to different tasks; the second network is often broader, more accessible to train, and capable of extracting finer features.

How beneficial the weights are; in other words, the network might be given instructions to better comprehend the intricacy of the image.

#### Convolutional layers

Although there may be convolutional layers, further convolutional layers, or pooling layers behind, the convolutional layer is the first layer in a neural network, and the fully connected layer is the final. With each increase complexity, CNN’s complexity rises, making it possible to recognize more images. On the primary layer, essential elements like colour and borders are accentuated.

This layer, known as the pooling layer, reduces the dimensionality of the Convoluted Format, which lowers the processing resources needed to analyses data through dimensionality reduction. Furthermore, extracting important characteristics independent of rotation and position increases the model’s capacity to be effectively trained. There are two different pooling techniques: max pooling and average pooling.

#### Batch normalization

Reducing internal covariate shift was the initial goal of batch normalizing. Deep networks are especially prone to this issue as even little changes in shallower hidden layers can result in considerable changes in deeper hidden layers as they spread across the network.

Convolutional to fully linked layers frequently utilize flattened layers to condense inputs from several scales to a single scale.

Another crucial component of CNN is the dropout layer. The dropout layer serves as a mask, maintaining all other neurons’ functioning but excluding specific neurons’ contribution to the subsequent layer. The input pattern can be applied to a dropout layer, in which case it also applies to layers that are not visible, invalidating some of the input pattern’s features. Although overfitting to the training data should be avoided, dropout layers are crucial in training CNNs.

A dense layer in a neural network is one whose initial few levels are closely linked, which means that each layer’s neurons are connected to those of every other layer. Artificial neural networks employ Internet networks most frequently at this layer. The model’s dense layer neurons multiply matrices and vectors and receive outputs from all the neurons in the layer above them. The Adaptive CNN architecture is depicted in Fig. 3.

Most activities in the context of incremental learning centre on using information from earlier tasks and transferring it to more recent ones. Little focus has been placed on the equally crucial problems of modernizing the model’s hardware and energy needs. This task’s central concept is a unique “copy and branch” method that enables the network to learn new tasks sequentially without sacrificing performance on earlier jobs. Replicated layers offer a solid starting point for understanding new jobs compared to randomly initialised weights. Education converges quicker as a result of the kernel’s rapid learning. Instead of starting over, you can fine-tune each new training season, saving time and effort. On the other hand, branches enable the network to retain task-specific weight parameters so that, regardless of how much it learns new tasks, it does not lose track of past tasks (in task-specific circumstances). To underline the significance of network sharing from a hardware standpoint, quantitatively measure the energy consumption, training time, and memory storage reductions associated with models trained with varied degrees of sharing. Our suggested technique is unique in that it can be implemented on existing hardware if it does not require any modifications to the algorithm and can allocate more RAM to the additional parameters necessary for learning new classes. There is no cost for the learned classes to keep data samples or statistics.

### Network architecture

Our suggested model, called Tree-CNN, is based on hierarchical classifiers and has numerous nodes linked in a tree-like manner. Each node has a DCNN that has been trained to categorise the input to one of its offspring (leaf nodes are an exception). The topmost node in the tree, or root node, is where the first categorization occurs. Based on the categorization label, the image is subsequently transmitted to the child nodes. Up to the leaf nodes, the final stage of classification, this node further categorises the images. Branch nodes are intermediate nodes with a parent node, and one or more child nodes. The tree’s last level is a leaf node. No two leaf nodes have the same class, which is individually linked with each leaf node, a two-level categorization network’s root and branch nodes. A leaf node, the output node of the fork CNN, is the output node of each second-level fork node.

### The learning algorithm

Assume that you already have a model that can recognize a specific number of things. The model may consist of several hierarchical CNNs or just one CNN serving as the root node for many leaf nodes. Learning to identify images from M new categories is the definition of a new task. To begin the root node of a particular is given model a tiny image sample (around 10%) from the fresh training set as input. New classes with a high chance are added to the Tree-CNN first when sorting is completed. Because Softmax modifies the output layer’s reaction to the image exponentially and aids in better identifying how similar the image is to one of the currently used labels, it should be used instead of the number of images classified into each child node.

**Figure 4:**
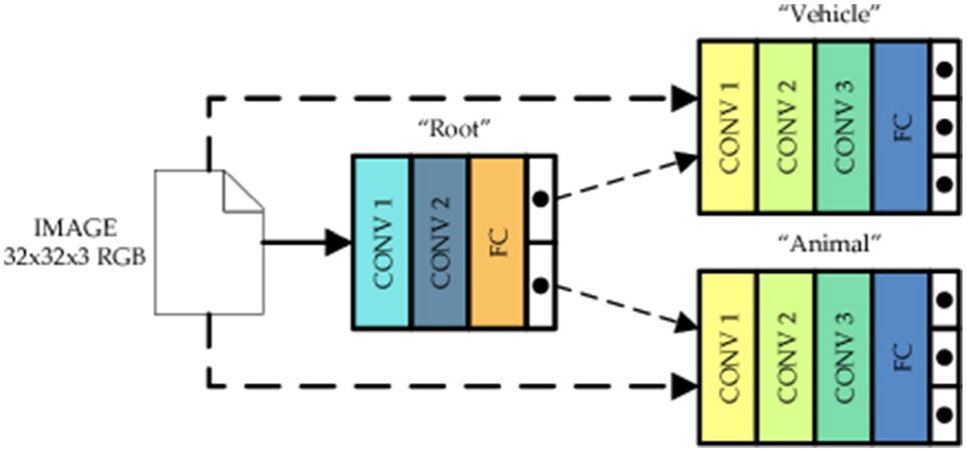
Proposed tree-CNN.

Once the M-type “Grow-Tree” method has been completed on the root node, it may advance to the following tree level to develop a deeper Tree-CNN model, as shown in Fig. 4. The child node where you wish to add the new class may now use the same procedure. The deputy director selects the tree’s planting method. According to the user’s limitations, the algorithm determines how to develop the tree. The modified/new node receives supervised training based on gradient descent whenever a new class is assigned to a place in the tree. As a result, the network’s impacted components need to be retrained or fine-tuned, rather than the entire network needing to be changed. The root node is trained on all available data at each incremental learning step since it must learn how to categories all objects into the next branch. A branch node only becomes active during inference when the root node arranges the input to that branch node. No matter how the branch nodes are categorized, it is still incorrect if the root node is misclassified. As a result, it only trains branch nodes that fall under the designated category. The branch node lookup table does not change throughout the incremental learning phase; it stays the same.

### Hybrid adaptive CNN with tree-CNN

Each object class in the user-accessible datasets has a different label. However, the root and branch nodes of Tree-CNN often combine, merge, or separate these classes in accordance with the needs of the algorithm. Each node in Tree-CNN keeps its own “LabelsTransform” lookup table to guarantee label consistency. The lookup table will be updated with the new class assigned to one of the root node’s existing output nodes, for instance, if it is added. The class label and new output node are also added as new entries in the lookup table whenever a new class is introduced as a new node. As each class is eventually connected to a distinct leaf node, leaf nodes do not need a lookup table. For the new class being evaluated, if two nodes are combined, the node with the lower average softmax value (node A) is combined with the one with the higher average softmax value (node B). If both softmax values are equal, a random selection is made. The class label formerly allocated to node A has been moved to node B at the root node level of the lookup table. The combined Node B lookup database adds these class labels from Node A as new entries and new leaf nodes with entries.

**Figure:**
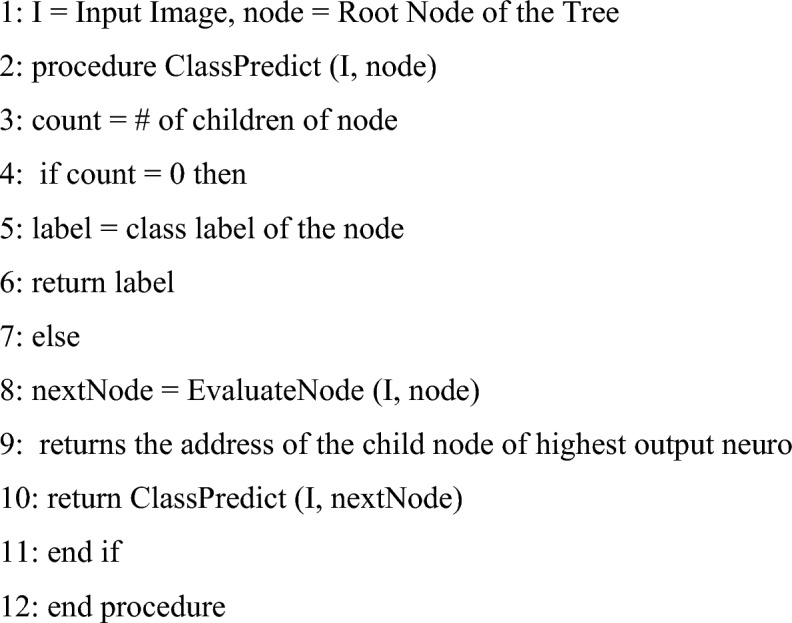
Algorithm:

## Results and discussion

Cancer morbidity and medical costs are exacerbated by the presence of malignant lesions. As a result, researchers have concentrated their efforts on developing algorithms that are very accurate and adaptable when identifying early indications of skin cancer. Early identification is crucial because malignant melanocyte cells spread, infiltrate, and multiply quickly^31^. Specialists routinely employ dermoscopy and epiluminescence microscopy (ELM) to determine whether a benign or malignant skin lesion.

In dermatology, a magnifying lens and light are used to observe medical patterns, including colors, veils, pigmented nets, globs, and ramifications more clearly^32,33^. People with visual impairments may notice morphological features that are normally concealed. These include the ABCD (Asymmetrical form, Border anomaly, Color discrepancy, Diameter, and Evolution)^34^, 7-point checklist^35^, and pattern analysis^30^. Non-professional dermoscopic images may predict melanoma with a 75–80% accuracy, although interpretation requires time and is highly individualised based on the dermatologist’s level of expertise^36^. Computer-aided diagnosis (CAD) methods have made it simpler to get beyond these obstacles^33,36^. Artificial Intelligence (AI) that is based on Deep Learning (DL) has significantly improved the ability to diagnose cancers^37,38^. Since dermatologists and laboratories are few in rural regions, automating the classification of skin lesions might aid in early diagnosis and screening for skin cancer^39,40^.

By using the Adam optimizer and learning speed strategies like validation tolerance—which slows down learning when it gets stuck for an extended amount of time—this dataset is utilized to pre-train the suggested framework. During the learning phase, the Adam optimizer receives the ensuing hyperparameters. The batch size increased to 64, which is double the previous figure of 2. It is the year 50. Ten is the patience factor. In this scenario, the momentum is 0.9. “Batching” is a mode of contagious transmission that completes our anti-infection defenses. To learn the suggested DL system, an 80% random image set is utilized. The correct weight combinations are saved for subsequent use in a validation set, which is made up of 10% of the training data after training. For every iteration, an adjustable learning rate is applied. The three models have dropout rates of 0.1, 0.15, and 0.2, respectively.

By splitting training and testing on the proposed system’s dataset from 90 to 10, some evaluation findings are provided. In order to cut down on the amount of time needed to finish the project, and made this divide. Fifty generations of models were trained with batch sizes ranging from 2 to 64 and learning rates ranging from 1 $\times$ 10^4^, 1 $\times$ 10^5^, and 1 $\times$ 10^6^, using 10% of the proposed adaptive CNN training set as the validation set. By freezing varying numbers of layers, the suggested adaptive CNN is further improved to achieve usable accuracy potentially. After that, a softmax layer at the conclusion of the model divides the input training images into seven groups. 224 × 224 pixels are the new size for RGB input images. The adaptive CNN model’s training phase uses subsamples from the dataset. For every subsampled data point, compute the error. If the error exceeds the threshold, remove the point and continue the training. Furthermore, all three models’ attributes are prioritized from highest to lowest. Features with zero variance are eliminated and forwarded to the subsequent layer.

### Dataset

Table 1 displays the distribution of skin cancer grades for dermoscopic images in different lesion datasets. It represents a collection of 1600, 1000, and 1600 images from ISIC-2019, PH-2, and ISIC-2017 for teaching and modelling. Based on these datasets, segmented the KNN-based skin cancer classification model, performed a comparison analysis using the FCEDN-SpaSA concept to confirm the effectiveness of the suggested model, and carried out the following sections by simulation findings for parameters like precision, sensitivity, FNR, prediction time, and others.

**Table 1:** Description of the data set for the intelligent classification model and automated skin lesion.

Class	ISIC-2019		PH-2		ISIC-2017	
	Training	Testing	Training	Testing	Training	Testing
Melanoma	700	1000	300	700	700	1000
Nonmelanoma	700	1000	300	700	700	1000

#### Performance metrics

Six criteria, precision, specificity, precision, F1-score, sensitivity, and Matthew’s correlation coefficient, were used to assess performance analysis (MCC). This measuring formula’s mathematical model is:

3 $$Specifity=\frac{TN}{TN+FP}\times 100,$$

4 $$Accuracy=\frac{TP+TN}{TP+TN+FN}\times 100,$$

5 $$Precision=\frac{TN}{TN+FP}\times 100,$$

6 $$Sensitivity=\frac{TN}{TN+FP}\times 100,$$

7 $$F1\,score=2\times \frac{Precision\times Sensitivity}{Precision+Sensitivity}\times 100,$$

8 $$MCC=\frac{TP\times TN-TP\times FN}{\sqrt{\left(TP+FP\right)\times \left(TP+FN\right)\times \left(TN+FP\right)\times \left(TN+FN\right)}}\times 100.$$

Here, TP and TN represent the number of pixels of correctly categorised backdrops and objects. FN and FP numbers correspond to the number of pixels assigned to background-designated items and objects, respectively.

#### Preprocessing

**Figure 5:**
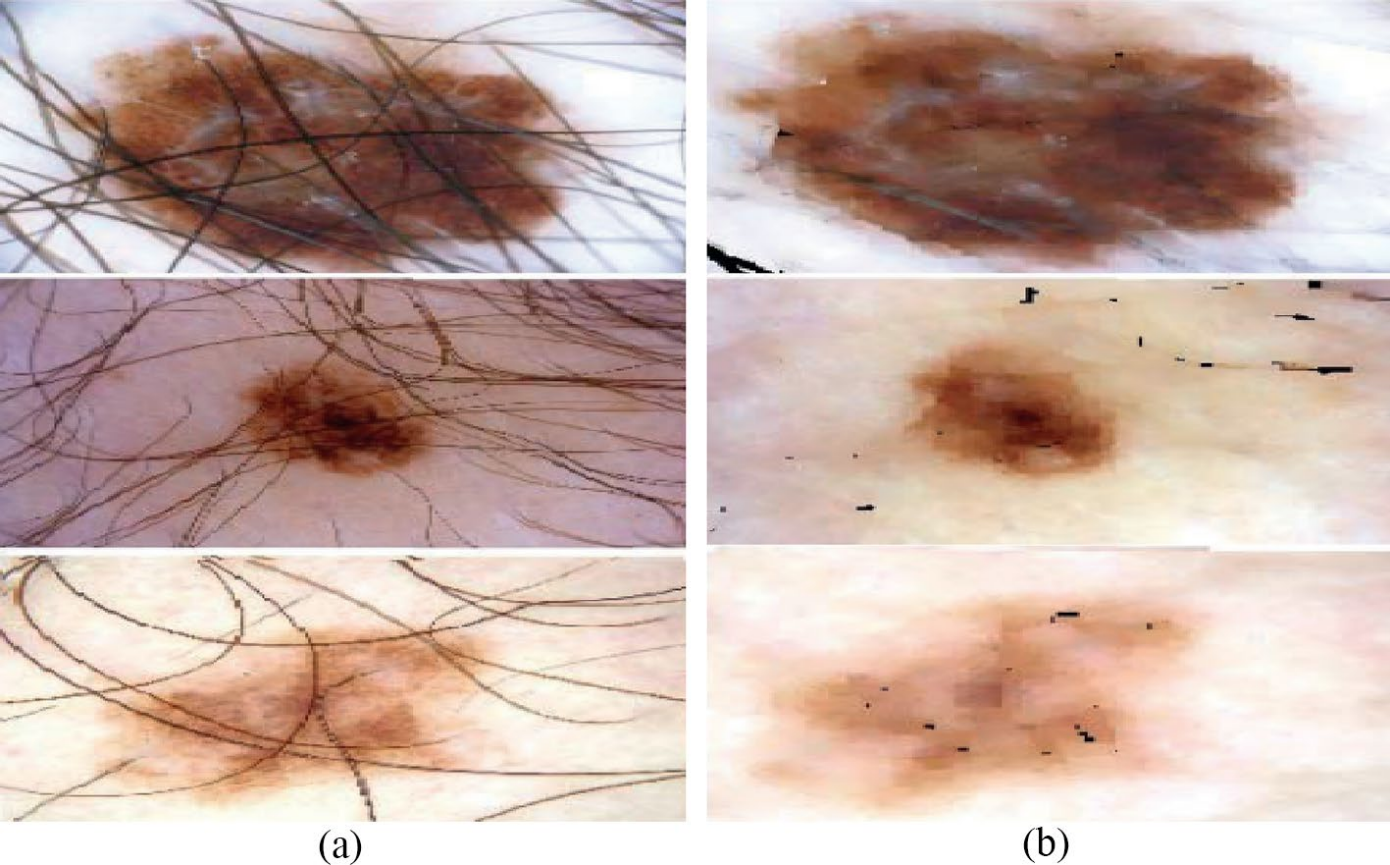
Original image (**a**) and preprocessed image (**b**) are the outcomes of preprocessing.

Dermoscopic images of skin lesions are preprocessed in two steps, the first being the most crucial. The depilatory notion is based on morphological adjustments that make choosing the right ROL easier. After the hair removal procedure, another pre-processing stage that benefits from intensity-based image enhancement. Improving the hairless image after preprocessing makes it easier to segment dermoscopic images using ROL accurately. The outcomes of the preprocessing procedure are displayed in Fig. 5.

#### Segmentation

**Figure 6:**
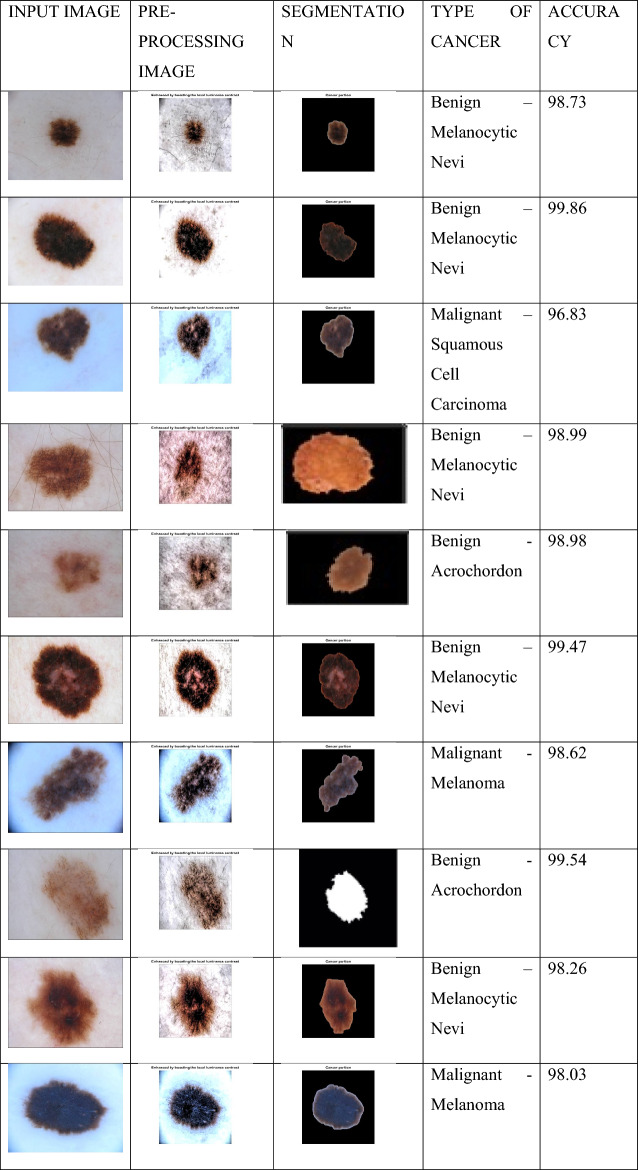
Proposed lesion location, with findings identified.

Here, the visually segmented image is shown together with the lesion segmentation number findings and compared with the state-of-the-art in terms of accuracy values. The results of the suggested lesion segmentation for a few datasets are shown in Table 2. The accuracy average of each image picked in a split was used to compute the results displayed in this table. The produced image is compared with the provided real image following segmentation using the newly built Adaptive CNN model. Every image that is added to the database is processed in the same way. The average accuracy, FNR, and overall running time for each dataset were then determined. The suggested segmentation approach in ELM yields an average accuracy of 95.28%, as shown in Table 2. The lesion segmentation inspection time was 51.3652 s, and the error rate was 4.69% (s). An accuracy of 95.89% and an error rate of 4.32% are again obtained using KELM. This dataset’s known test time is 59.5160 (s). Another challenging split, MSVM, attained an accuracy of 92.70%. The execution time is 67.4202 (s), and the error rate is 7.45%. Finally, presented the proposed FCEDN-functionality. SpaSA’s with a 1.5% error rate 98.78% accuracy were attained. The execution took 29.3456 s. As a consequence, it is apparent that the execution time grows longer as the dataset size rises. For instance, FCEDN-SpaSA required just 29.3456 (s) for 100 images. Figure 6 shows the identification of different types of skin cancer and their accuracies. The MATLAB R2018a was used for the skin cancer prediction.

**Table 2:** Accuracy of the proposed lesion segmentation method by employing the contrast enhancement approach.

Optimization	Calculated measures		
	Accuracy (%)	Error (%)	Testing time (s)
ELM	95.28	4.69	51.3652
KELM	95.89	4.32	59.5160
MSVM	92.70	7.45	67.4202
FCEDN-SpaSA	98.78	1.5	29.3456

In the segmentation result, the proposed method of accuracy classification accuracy was the greatest at 98.42% using simulations with the PH-2 data set. From Table 3, the proposed FCEDN-SpaSA segmentation of JAC and DIC have accuracy rates of 90.14% and 94.76%, respectively.

**Table 3:** Results of proposed FCEDN-SpaSA segmentation metrics (%) on the PH2 dataset.

References	Year	JAC	DIC
K means clustering	2022	81.46	88.25
Modified K means clustering	2022	85.98	92.51
Modified AD-GLCM segmentation	2022	89.46	94.64
CDNN	2023	88.63	93.93
Proposed FCEDN-SpaSA	2023	90.14	94.76

In the segmentation result, the proposed accuracy method was the greatest at 97.65% using simulations with the ISIB2017 data set. From Table 4, the proposed FCEDN-SpaSA segmentation of JAC and DIC have accuracy rates of 92.56% and 95.43%, respectively.

**Table 4:** Results of Proposed FCEDN-SpaSA segmentation metrics (%) on the ISIB2017 dataset.

References	Year	JAC	DIC
K means clustering	2022	76.30	84.27
Modified K means clustering	2022	77.16	87.40
Modified AD-GLCM segmentation	2022	78.89	88.76
CDNN segment	2023	80.45	87.23
Proposed FCEDN-SpaSA	2023	92.56	95.43

In the segmentation result, the proposed method of average classification accuracy was the greatest at 97.65% using simulations with the ISIB2017 data set. From Table 5, the proposed FCEDN-SpaSA segmentation of JAC and DIC have accuracy rates of 91.75% and 94.32%, respectively.

**Table 5:** Results of Proposed FCEDN-SpaSA segmentation metrics (%) on the ISIC 2019 dataset.

References	Year	JAC	DIC
Modified AD-GLCM segmentation	2022	78.88	83.73
CDNN segment	2023	84.51	87.64
Proposed FCEDN-SpaSA	2023	91.75	94.42

#### Classification

As a result of our calculations utilizing the suggested framework, the numbers are displayed in Table 6. The proposed framework employed the Adaptive CNN classifier. For comparison, used the Naive Bayes, ELM, MSVM, and KELM classifiers. The table shows that Adaptive CNN achieved 91.67% accuracy and 9.43% FNR in a record-breaking amount of time, 133.4632 (s). With a FNR of 14.34%, a time of 121.5230, and the second-highest accuracy of 85.45%, MSVM comes in second (s). Although MSVM performs better than Adaptive CNN during the test, the difference between the two is considerable. Accuracy values for Naive Bayes, ELM, and KELM are 82.34%, 83.23%, and 82.45%, respectively.

**Table 6:** Performance analysis of classification.

Classifier	Performance measures			
	Accuracy (%)	Sensitivity (%)	FNR (%)	Prediction time (s)
Navie Bayes	82.34	81.24	18.23	157.2304
ELM	83.23	83.15	15.12	138.3352
MSVM	85.45	85.23	14.34	121.5230
Adaptive CNN	91.67	91.27	9.43	133.4632

A detailed comparison of the state-of-the-art methods employing the PH2, ISBI-2017, and ISIC 2019 data sets can be found in Tables 7, 8 and 9. Using the suggested method, the classification accuracy of all datasets is maximized. Using color and texture features, the maximum classification accuracy on the PH2 dataset was 93.45% in the ResNet50; however, with the proposed adaptive CNN method, it was demonstrated to be 98.42%. The accuracy of the proposed work of the ISIB 2017 dataset is 97.65%, and the ISIB 2019 dataset is 98.09%. This demonstrates that the suggested adaptive CNN classifier model produces far superior results than the conventional algorithm, and the outcomes indicate that the adaptive CNN classifier classification technique is more reliable and effective.

**Table 7:** State of the art comparison of the Adaptive CNN classifiers on PH2 dataset.

Classifier	Accuracy (%)	Sensitivity (%)	Specificity (%)	AUC	Time (in ms)
Machine learning					
TREE	74.86	74.32	73.65	0.83	16.17
LR	77.42	78.53	76.97	0.86	14.73
SVM	96.21	95.73	97.65	0.89	11.52
LDA	91.89	90.04	92.41	0.88	13.45
KNN	92.15	92.18	92.43	0.90	12.24
DT	91.34	90.35	91.75	0.90	12.80
ANN + GWO	96.82	96.04	97.17	0.94	9.27
SVM + PSO	96.43	97.16	96.32	0.95	6.49
ANN + IGWO	96.62	97.52	96.15	0.96	6.18
Deep learning					
AlexNet	91.34	91.13	90.89	0.91	6.54
GoogLeNet	92.56	91.67	92.10	0.92	5.86
ResNet50	93.45	92.90	93.87	0.92	5.10
Adaptive CNN (proposed)	98.42	97.56	97.49	0.99	4.19

**Table 8:** State of the art comparison of the Adaptive CNN classifiers on the ISIB 2017 dataset.

Classifier	Accuracy (%)	Sensitivity (%)	Specificity (%)	AUC	Time (in ms)
Machine learning					
TREE	74.86	74.32	73.65	0.85	16.17
LR	77.42	78.53	76.97	0.87	14.73
SVM	94.21	95.73	95.65	0.92	11.52
LDA	91.89	90.04	92.41	0.89	13.45
KNN	92.15	92.18	92.43	0.91	12.24
DT	91.34	90.35	91.75	0.89	12.80
ANN + GWO	96.82	96.04	97.17	0.95	9.27
SVM + PSO	96.43	97.16	96.32	0.96	6.49
ANN + IGWO	96.62	97.52	96.15	0.96	6.18
Deep learning					
AlexNet	92.54	92.37	91.78	0.92	8.14
GoogLeNet	93.76	93.54	93.57	0.93	7.67
ResNet50	93.98	93.94	93.87	0.93	6.95
Adaptive CNN (proposed)	97.65	97.98	97.12	0.99	4.19

**Table 9:** State of the art comparison of the Adaptive CNN classifiers on the ISIB 2019 dataset.

Classifier	Accuracy (%)	Sensitivity (%)	Specificity (%)	AUC	Time (in ms)
Machine learning					
TREE	75.98	74.88	74.71	0.86	18.26
LR	78.57	78.65	77.78	0.89	15.47
SVM	95.76	94.16	95.59	0.93	13.61
LDA	94.14	94.49	94.78	0.88	14.67
KNN	93.60	94.82	94.70	0.90	13.82
DT	92.41	93.81	93.14	0.90	13.82
ANN + GWO	96.90	95.35	95.49	0.94	8.70
SVM + PSO	96.78	96.26	96.70	0.95	7.48
ANN + IGWO	97.87	97.91	97.82	0.96	5.14
Deep learning					
AlexNet	93.18	92.75	93.83	0.93	9.74
GoogLeNet	93.65	93.48	93.82	0.94	8.16
ResNet50	94.16	93.83	94.94	0.94	6.40
Adaptive CNN (proposed)	98.09	97.92	98.43	0.99	4.19

A comparison of efficacy to different strategies is made. According to Table 4, our method performs better than other methods regarding efficiency and effectiveness. Overall, the suggested inception model outperforms the existing approaches with an accuracy rate of almost 98 percent. It may thus be expanded to include the potential assessment of additional medical images (Table 10).

**Table 10:** Comparison with other deep learning techniques to detect skin cancer.

References	Dataset	Model	Accuracy (%)
^38^	HAM10000	RegNetY-3.2GF	85.8
^41^	HAM10000	AlexNet	84
^42^	HAM10000	MobileNet	83.9
^43^	ISIC2019	CNN	83.1
^43^	ISIC2017	Resnet-50	83.6
^44^	HAM10000	MobileNet, VGG-16	80.61
^45^	ISIC2017	Resnet-50	85
^46^	HAM10000	Support Vector Machine (SVM), Logistic Regression (LR), Random Forest (RF), AdaBoost (Adaptive Boosting), Balanced Bagging (BB) and Balanced Random Forest (BRF)	74.75
^47^	HAM10000	CNN	77
^48^	HAM10000	ResNet	78
		Xception	82
		DenseNet	82
^49^	HAM10000	MobileNet and LSTM	85
^50^	HAM10000	CNN	86
^50^	HAM10000	Modified Resnet-50	85.3

## Conclusion

Skin cancer specialists can manually identify malignant spots using dermoscopy images, but this is still a challenging effort. Hence automated approaches were created to make the procedure easier. The dataset categorised skin cancer images in this work using the ISIC-2017, ISIC-2019, and PH-2 databases. The preprocessing methods required to create an automated skin cancer detection system are described in this article. Image enhancement and restoration are the two elements that split the entire procedure. Both processes include detailed descriptions of all the stages involved in practical methods for enhancing skin cancer images and applicable filters for image noise removal and image smoothing. Using dynamic microscope imaging, a SpaSA-based hyperparameter optimised FCEDN was created to identify skin cancer spots of interest. A Comparison of the effectiveness of SpaSA with four distinct Navie bays, ELM, and MSVM-based hyperparameter-optimized splits. In contrast to conventional approaches, the results demonstrate enhanced performance. For precise segmentation, the contrast expansion method specifically increases segmentation accuracy. Computation time is one of our work’s drawbacks; however, plan to solve it in subsequent efforts. The future work of this work are (1) to enhance prediction performance and suggest a hybrid approach built on deep learning and machine learning, (2) to combine several data-enhancement strategies to boost prediction accuracy, and (3) to examine the results in various learning contexts, including transitional and active learning.

## Data Availability

The datasets generated and/or analysed during the current study are available in the ISIC 2017–2018 and PH-2 repository, https://challenge.isic-archive.com/data/; https://www.fc.up.pt/addi/ph2%20database.html.
